# Effects of extreme precipitation on hospital visit risk and disease burden of depression in Suzhou, China

**DOI:** 10.1186/s12889-022-14085-w

**Published:** 2022-09-09

**Authors:** Gang Jiang, Yanhu Ji, Changhao Chen, Xiaosong Wang, Tiantian Ye, Yuhuan Ling, Heng Wang

**Affiliations:** 1 Department of Social Medicine and Health Management, School of Health Management, Anhui Medical University, Hefei, China; 2 Department of Epidemiology and Health Statistics, School of Public Health, Anhui Medical University, Hefei, China; 3 Department of Psychiatry, Suzhou Second People’s Hospital, Suzhou, China; 4 The First Affiliated Hospital of Anhui Medical University, Hefei, China

**Keywords:** Depression, Extreme precipitation, Time-series analysis, Disease burden

## Abstract

**Background:**

The purpose of this study was to explore the impact of extreme precipitation on the risk of outpatient visits for depression and to further explore its associated disease burden and vulnerable population.

**Methods:**

A quasi-Poisson generalized linear regression model combined with distributed lag non-linear model (DLNM) was used to investigate the exposure-lag-response relationship between extreme precipitation (≥95th percentile) and depression outpatient visits from 2017 to 2019 in Suzhou city, Anhui Province, China.

**Results:**

Extreme precipitation was positively associated with the outpatient visits for depression. The effects of extreme precipitation on depression firstly appeared at lag4 [relative risk (RR): 1.047, 95% confidence interval (CI): 1.005–1.091] and lasted until lag7 (RR = 1.047, 95% CI: 1.009–1.087). Females, patients aged ≥65 years and patients with multiple outpatient visits appeared to be more sensitive to extreme precipitation. The attributable fraction (AF) and numbers (AN) of extreme precipitation on outpatient visits for depression were 5.00% (95% CI: 1.02–8.82%) and 1318.25, respectively.

**Conclusions:**

Our findings suggested that extreme precipitation may increase the risk of outpatient visits for depression. Further studies on the burden of depression found that females, aged ≥65 years, and patients with multiple visits were priority targets for future warnings. Active intervention measures against extreme precipitation events should be taken to reduce the risk of depression outpatient visits.

**Supplementary Information:**

The online version contains supplementary material available at 10.1186/s12889-022-14085-w.

## Background

Depression is a common psychiatric disorder worldwide, characterized by sustained grief and a lack of interest or pleasure in activities that were previously beneficial or pleasurable [1]. As of 2019, approximately 280 million people worldwide are suffering from depression [2]. Depression has become the leading cause of disability worldwide and is a major contributor to the global burden of disease [3]. Therefore, it is of great importance to identify depression and the associated risk factors.

As is known to all, the risks for depression are both genetically and environmentally determined [4]. The risk of depression is partly mediated by genetic factors, accounting for less than 40% [5]. This suggests that environmental factors play an important role in the onset and development of depression. Some epidemiological evidence has shown that meteorological factors are associated with mental illness [6]. Scholars have studied the effects of meteorological factors such as sunshine, rainfall, temperature and pressure on the occurrence and admission of depression [7–9]. In particular, with the advancement of climate change, extreme weather events have further increased, and the impact of extreme weather events on mental diseases (such as depression, schizophrenia, bipolar disorder, etc.) has begun to be paid more attention [10]. Floods and rainstorms are gradually taken into account whether they are associated with depression [11, 12]. However, studies had found that the relationship between precipitation and depression were inconsistent. Some studies reported that precipitation can increase the risk of depression [7] or that it was a protective factor for depression [9]. While others discovered that there were no statistical significance effects between precipitation and depression [13–17]. Furthermore, no studies have investigated the impact of extreme precipitation on depression.

Considering the current state of research on depression, our research has three purposes: First, to explore the relationship between extreme precipitation and outpatient visits for depression. The second is to conduct subgroup analysis according to gender, age and visit types (first visit, multiple visits) to identify susceptible groups. The third is to assess the attributable burden of outpatient visits for depression due to extreme precipitation.

## Methods

### Study area

**Fig. 1:**
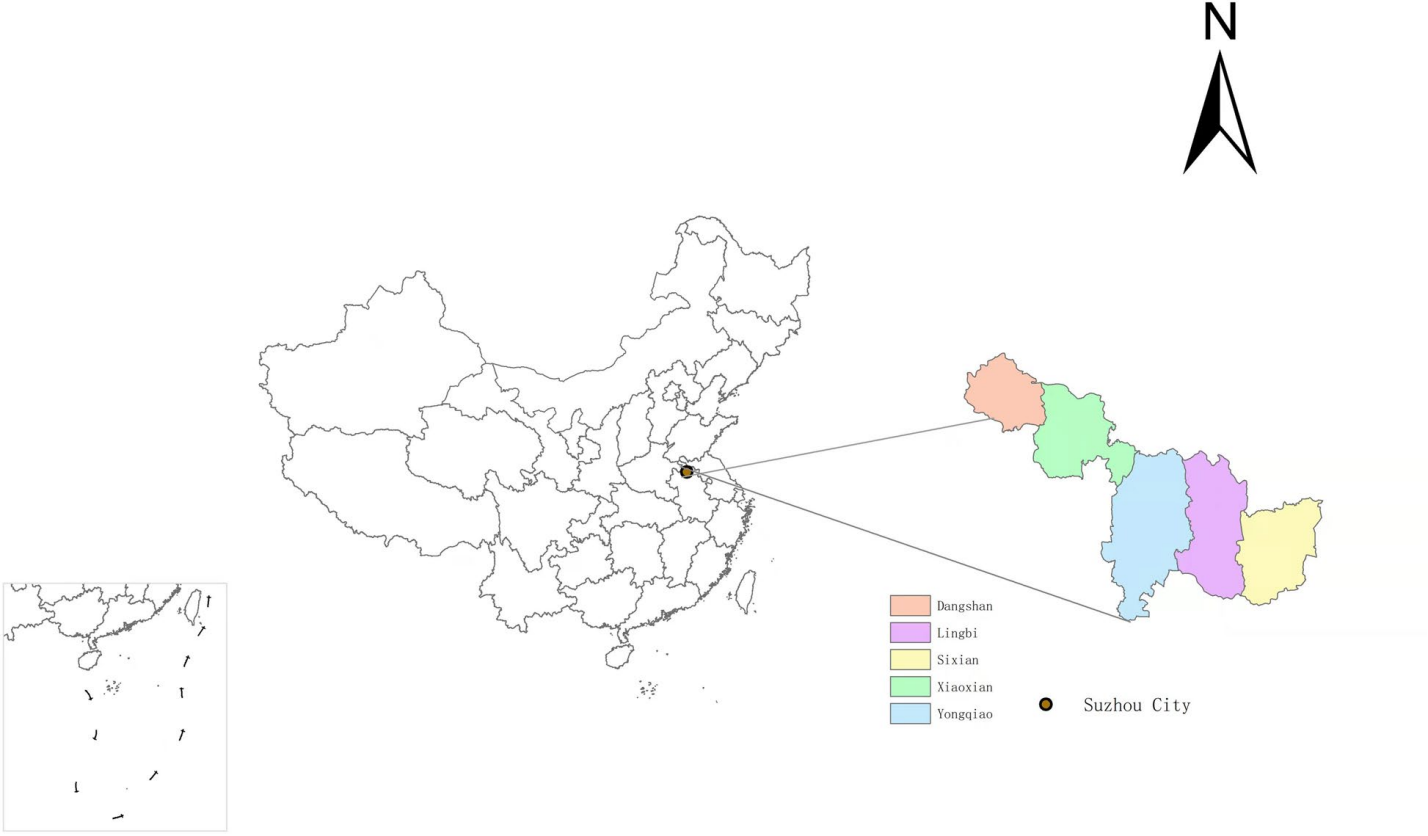
The geographical information of Suzhou, China

Suzhou is located in the northern Anhui Province, in the Yangtze River Delta and is known as the northern gate of Anhui Province. It lies between 116°09′-118°10′ east longitude and 33°18′-34°38′ north latitude, with a total area of 9939 km^2^. In 2020, Suzhou has a permanent population of 5,324,476 people. It’s a warm temperate semi-humid monsoon climate zone, and the main characteristics of Suzhou are mild climate, four distinct seasons, sufficient sunshine and moderate rainfall. Figure 1 presented the geographical location information of Suzhou.

### Data collection

In this study, daily depression cases from January 1, 2017, to December 31, 2019, were obtained from Suzhou Second People’s Hospital (Suzhou Mental Health Center), whose diagnosis and treatment of depression have a good credibility. The diagnosis of depression was based on the International Classification of Diseases, 10th edition (ICD-10 code: F32-F33). Case information includes gender, age, outpatient visits date, residential address, and visit types. Patients whose residential addresses were not in Suzhou were excluded.

Meteorological data, including daily mean temperature, rainfall, relative humidity, as well as sunshine duration, were obtained from China Meteorological Data Sharing Service System (http://data.cma.cn/). Daily air pollution data including particulate matter with aerodynamic diameter less than 2.5 μm (PM_2.5_), nitrogen dioxide (NO_2_) and sulfur dioxide (SO_2_) were retrieved from China National Environmental Monitoring Centre (http://www.cnemc.cn/).

So far, there is no unified description of the concept of extreme precipitation. In view of the regional and seasonal differences of precipitation distribution, extreme precipitation was defined by using the percentile method, which was also the method applied by many scholars [18, 19]. By using the 95th percentile as the cutoff points, we divided precipitation into three categorical variables, namely no precipitation (equal to 0 mm), moderate precipitation (> 0 mm and < 95th percentile) and extreme precipitation (≥95th percentile) [20].

### Statistical analysis

Previous studies have shown that the DLNM can better evaluate the nonlinear and delayed effects of environmental exposure on health outcomes [21]. Therefore, we performed a quasi-Poisson generalized linear regression model combined with DLNM to quantitatively access the impact of extreme precipitation on outpatient visits for depression. Potential confounding factors including long-term trends and seasonality, weekdays (DOW), public holiday (Holiday), daily mean temperature (MT), relative humidity (RH), and sunshine duration (SD) were included in the model. The model was shown as follows:

$${\displaystyle \begin{array}{c} Yt\sim quasi- Poisson\ \left({\mu}_t\right)\\ {} Log\ \left({\mu}_t\right)=\propto +{\beta EP}_{t,l},4+ ns\left( MT,3\right)+ ns\left( RH,3\right)\\ {}+ ns\left( SD,3\right)+ ns\left( Time,7\right)+\eta {DOW}_t+\gamma {Holiday}_t\end{array}}$$

In the formula, *t* represented the observation time (day); μ_t_ was the expected number of depression outpatient visits on day t; ∝ meant the intercept of the model; *β* was the cross-basis matrix coefficient produced by DLNM; *EP*_*t*, *l*_ referred to the extreme precipitation on day t; *l* was the number of lag days. In our study, extreme precipitation and lagged effects were comprised using a “natural cubic spline-natural cubic spline” method [22], and the degree of freedom (*df*) for exposure and lag dimensions are set to 1 and 4, respectively [23]. The ns() represented the natural cubic splines. Ns with 7 *df* per year was used to control long-term trend and seasonality. And ns with 3 *df* were used to accommodate the delayed effects of MT (lag 0–14), RH (lag 0–14) and SD (lag 0–14) [20]. Holiday and DOW were also controlled in the model as binary and categorical variables, respectively. The effect estimates were calculated as extreme precipitation relative to no precipitation.

According to the minimum Akaike Information Criterion (AIC), we selected 14 days as the maximum lag days to capture the effect of extreme precipitation (Table S1). Furthermore, subgroup analysis was performed to identify the susceptible population of depression caused by extreme precipitation based on gender (male, female), age (≤18 years, 19–39 years, 40–64 years and ≥ 65 years) and visit types (first visit, multiple visits). Those with two or more outpatient visits were considered as multiple visits. The statistical significance of the differences between subgroups was identified by calculating the 95% confidence interval (CI) of the formula [23]: $\left(\hat{{\mathcal{Q}}_1}-\hat{{\mathcal{Q}}_2}\right)\pm 1.96\sqrt{{\left(\hat{SE_1}\right)}^2+{\left(\hat{SE_2}\right)}^2}$, Where $\hat{{\mathcal{Q}}_1}$ and $\hat{{\mathcal{Q}}_2}$ were the estimates for the two groups, and $\hat{SE_1}$ and $\hat{SE_2}$ were their respective standard errors [24].

Attributable risk can better reveal disease burden of depression caused by exposure to extreme precipitation. In our study, we used the following formulae to calculate AF and AN, which can assess the burden of depression caused by extreme precipitation [23].

$${\displaystyle \begin{array}{c}A{F}_t=R{R}_t-1/R{R}_t\\ {}A{N}_t=A{F}_t\ast {N}_t\end{array}}$$

In the formulae, N_*t*_ meant the number of outpatient visits for depression on day *t*. AF represented the ratio of the number of depression cases attributed to extreme precipitation to the number of depression outpatient visits.

The “splines” and “dlnm” packages were used in R software (version 4.1.2) to perform all statistical analysis. Two-sided *P* values less than 0.05 were considered statistically significant.

### Sensitivity analysis

In this study, four sensitivity analyses were performed to test the robustness of the results: (1) changing the *df* for MT (3–6), RH (3–6) and SD (3–6); (2) varying the *df* (5–8) for time to adjust for long-term trend and seasonality; (3) replacing the P95 cut off value with different percentiles (P90, P92.5, P97.5 and P99) to check the stability of the model. (4) Air pollutants such as PM_2.5_, NO_2_, and SO_2_ have been shown to be associated with the risk of outpatient visits for depression [25], we compared the results before and after adding air pollutants to the model to test its robustness.

## Results

### Descriptive analysis

There were 26,343 depression cases from Suzhou during 2017–2019, with the daily average of 24.1 cases. Table 1 reflected the summary statistics of depression outpatient visits and environmental factors in 2017–2019. In terms of gender, there were more males than females, with male-to-female ratio being 1.5:1 (15,844:10499). In the age group, the highest proportion of cases were in the 40–64 years age group (accounting for 41.3%), 8.3% in the ≤18 years age group, 30.7% in the 19–39 years age group and 19.6% in the ≥65 years age group. The average values of daily rainfall, mean temperature, relative humidity and sunshine duration were 2.4 mm, 15.8 °C, 73.6% and 5.8 h, respectively. The daily average concentrations of air pollutants were 58.0 μg/m^3^ for PM_2.5_, 34.5 μg/m^3^ for NO_2_ and 13.6 μg/m^3^ for SO_2_, respectively. The maximum daily rainfall was 232.6 mm during the study period.

**Table 1:** Descriptive statistics of depression outpatient visits and environmental factors in Suzhou, 2017–2019

Variables	Total	Mean ± SD	Min	*P*25	Median	*P*75	Max
Depression cases	26,343	24.1 ± 8.2	4	19	25	30	40
Gender							
Female	10,499	9.6 ± 4.2	0	7	10	13	21
Male	15,844	14.5 ± 5.5	4	10	12	20	21
Age							
≤ 18 years	2198	2.0 ± 2.4	0	0	1	3	8
19–39 years	8083	7.4 ± 2.6	0	6	7	9	15
40–64 years	10,889	9.9 ± 2.5	2	9	10	12	17
≥ 65 years	5173	4.7 ± 3.3	0	1	4	8	9
Visit types							
First visit	12,567	11.5 ± 5.5	1	7	9	17	18
Multiple visits	13,776	12.6 ± 4.2	2	10	13	16	24
Weather conditions							
Rainfall (mm)	/	2.4 ± 10.7	0.0	0.0	0.0	0.0	232.6
Mean temperature (°C)	/	15.8 ± 9.9	−6.3	6.8	16.3	24.7	34.4
Relative humidity (%)	/	73.6 ± 13.9	27.0	64.0	75.0	84.0	99.0
Sunshine duration (h)	/	5.8 ± 4.3	0.0	0.8	7.1	9.4	12.8
Air pollutants							
PM_2.5_ (μg/m^3^)	/	58.0 ± 36.7	0.0	32.0	49.0	75.0	250.0
NO_2_ (μg/m^3^)	/	34.5 ± 17.9	5.0	22.0	31.0	45.0	121.0
SSO_2_ (μg/m^3^)	/	13.6 ± 8.1	3.0	8.0	12.0	17.0	70.0

*SD* Standard deviation, *P25 P75* the 25th percentile, the 75th percentile, *Min* Minimum, *Max* Maximum

**Fig. 2:**
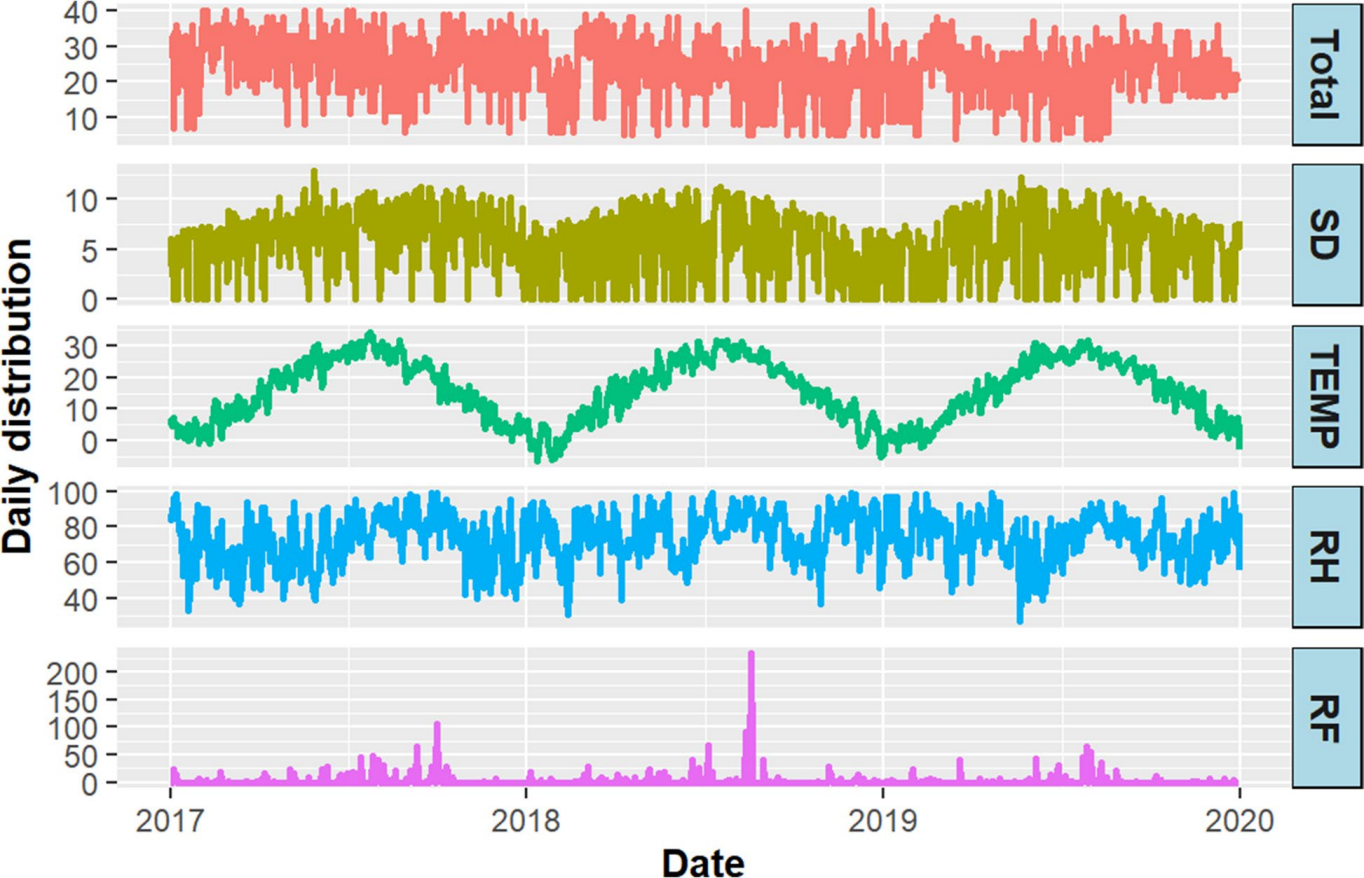
The time-series distribution of daily depression outpatient visits and weather factors in Suzhou, 2017–2019. TEMP Temperature; RH Relative humidity; RF Rainfalls; SD Sunshine duration

The time-series distribution of daily depression outpatient visits and weather factors in Suzhou from 2017 to 2019 were shown in Fig. 2. There was a distinct seasonality in the distribution of temperature, relative humidity, rainfall and sunshine duration.

### Association between extreme precipitation and outpatient visits for depression

**Fig. 3:**
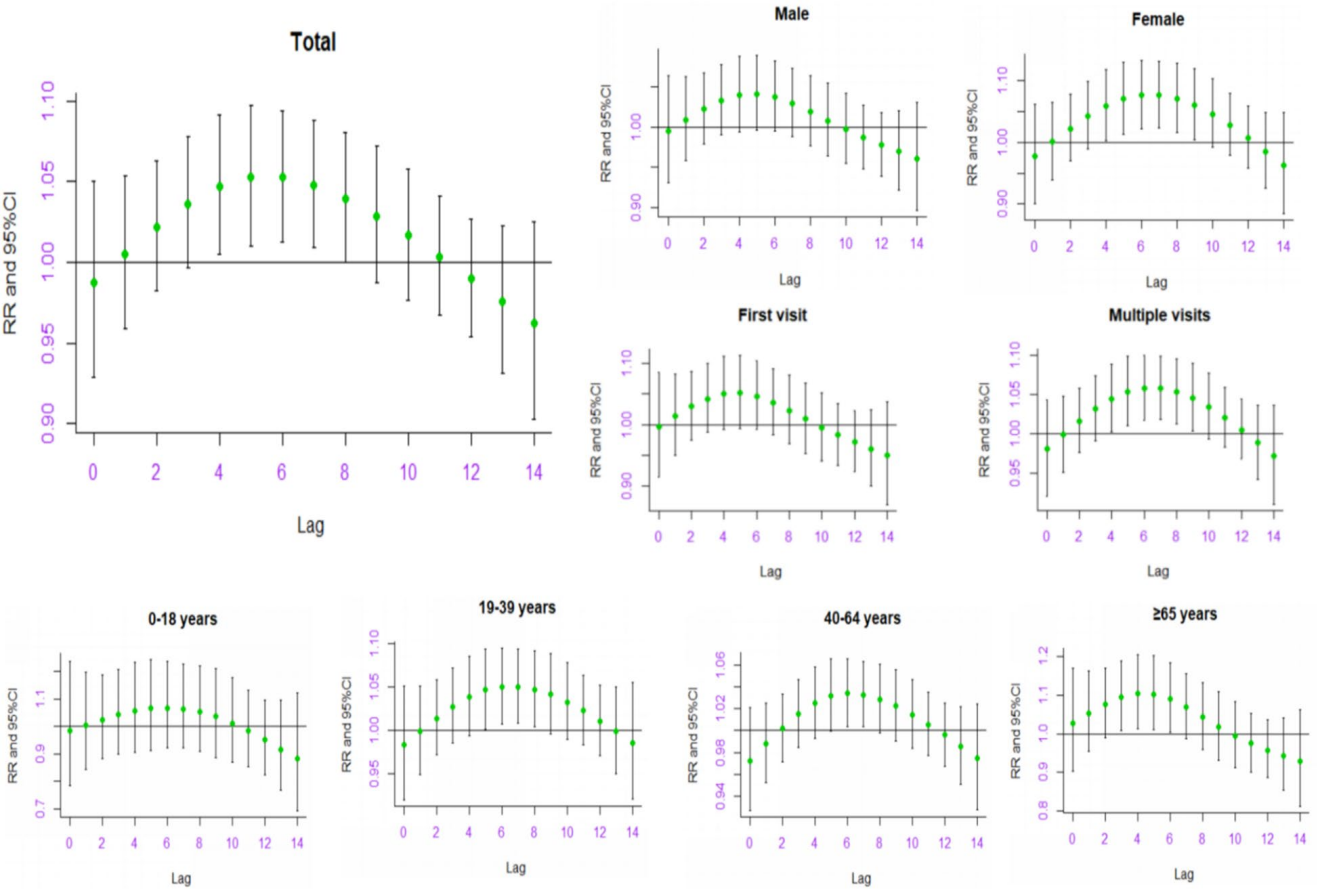
The relative risk (RR) and 95% confidence interval (95% CI) of extreme precipitation on total and subgroups (gender/age/visit type) outpatient visits for depression in diverse lag days

Figure 3 showed the RR and 95% CI of extreme precipitation on total and subgroups (gender/age/visit type) outpatient visits for depression in diverse lag days. We found that the association between extreme precipitation and outpatient visits for depression was significant from lag4 (RR = 1.047, 95% CI: 1.005–1.091) to lag7 (RR = 1.047, 95% CI: 1.009–1.087), with the strong effect occurred at lag5 (RR = 1.052, 95% CI: 1.010–1.096). In gender subgroup analysis, we observed that significant effects on female rather than male. For female, extreme precipitation effect on outpatient visits for depression occurred at lag4 (RR = 1.059, 95% CI: 1.002–1.118), lag5 (RR = 1.070, 95% CI: 1.013–1.130), lag6 (RR = 1.076, 95% CI: 1.022–1.133), lag7 (RR = 1.076, 95% CI: 1.023–1.131), lag8 (RR = 1.070, 95% CI: 1.016–1.127) and lag9 (RR = 1.060, 95% CI: 1.004–1.120). In term of age and visit type group, we found that people older than 65 years and multiple visits cases were more sensitive to extreme precipitation (Table S2).

### Attributable risk of extreme precipitation for depression

Table S3 displayed the single-day and cumulative lag effects of extreme precipitation on depression outpatient visits at various lag days in Suzhou, China. Based on several published studies [20, 23], we chose the maximum single-day lag effect [lag5, 1.052(1.010–1.096)] to calculate the corresponding AF and AN in our study. Table 2 displayed the AF and AN of extreme precipitation on outpatient visits for depression. The AF and AN of extreme precipitation on outpatient visit for depression were 5% and 1318.25, respectively. In subgroups analysis, we found that the AF and AN of female cases were 6.58% (95% CI: 1.33–11.56%) and 691.29, respectively, which were both higher than male cases [AF: 3.94% (95% CI: − 0.43–8.13%); AN: 624.83)]. The results of age stratification indicated that cases aged ≥65 years group [AF: 9.37% (95% CI: 1.11–16.94%); AN: 484.93] were more susceptible than all other age groups. In term of visit types, cases with multiple visits had a high disease burden [AF: 5.04% (95% CI: 1.01–8.91%); AN: 694.54]. Those indicated that females, aged ≥65 years and with multiple visits had a higher disease burden due to extreme precipitation (Fig. S1).

**Table 2:** Attributable fractions (95%CI) and number of depression outpatient visits stratified by gender, age and visit types in Suzhou, 2017–2019

Group	AN	AF	95% CI	
Total	1318.25	5.00%	1.02%	8.82%
Male	624.83	3.94%	−0.43%	8.13%
Female	691.29	6.58%	1.33%	11.56%
0–18 years	135.31	6.15%	−9.58%	19.63%
19–39 years	359.88	4.45%	0.12%	8.59%
40–64 years	333.75	3.06%	−0.09%	6.12%
≥ 65 years	484.93	9.37%	1.11%	16.94%
First visit	618.12	4.92%	−0.66%	10.18%
Multiple visits	694.54	5.04%	1.01%	8.91%

*AF* Attribution fraction, *AN* atTribution number, *CI* Confidence interval

### Sensitivity analysis

When altering the *df* for time (5–8), mean temperature (3–6), relative humidity (3–6) and sunlight duration (3–6), we found that there were no significant difference on the effects of extreme precipitation on outpatient visits for depression (Fig. S2-S5). Moreover, there was little change before and after adding the pollutants (PM_2.5_, NO_2_ and SO_2_) to the model, which indicated that our results are robust (Fig. S6). At last, by changing the cut-off values of extreme precipitation from P95 to P90, P92.5, P97.5 and P99, we discovered no significant changes in the effect estimates values (Fig. S7).

## Discussion

In recent years, depression has been increasingly recognized as a major health problem, and climate change may exacerbate the burden of depression [26]. In this study, we examined the impact of extreme precipitation on outpatient visits for depression. And the results showed that extreme precipitation may increase the risk of depression outpatient visits, with significant effects lasting from lag4 to lag7. We further assessed the burden of depression caused by extreme precipitation and found that the AF and AN were 5.00%(95% CI: 1.02–8.82%)and 1318.25, respectively. Besides, female, cases aged ≥65 years and patients with multiple visits appeared to be more susceptible to extreme precipitation.

There is a clear lack in studies on depression and precipitation, and the results varied across different studies. As early as 1996, studies by Molin et al. showed that rainfall was not associated with the onset of winter depression [13]. Subsequently, studies in the Netherlands, Canada, North America and Sweden also showed that there was no relationship between rainfall and the occurrence of depression [14–17]. In our study, we suggested that extreme precipitation was associated with increasing outpatient visits for depression, which was similar with the results of Hare et al. [7] and Abbasi et al. [27]. But Hare used the linear regression models to estimate the effect of climate on depression, and annual cumulative rainfall index only reflected the long-term impact of rainfall on the occurrence of depression rather than daily effect [7]. In Abbasi’s study, the author took the rainfall regime and the behavior of rainy seasons into more account rather than precipitation itself [27]. In 2014, a cohort study of Spanish college graduates using cox regression models to assess the relationship between climatic factors and depression, and found that men who lived in rainy areas had a lower risk of developing depression [9]. These results were inconsistent with the conclusion of our study, which may be due to the fact that our study focused on the impact of extreme precipitation. In addition, different study areas, study designs and statistical methods may also lead to different conclusions [28, 29]. In view of the inadequacy and inconsistency of previous studies, further research is necessary to explore the association between extreme precipitation and the outpatient visits for depression.

Previous studies have explored the biological mechanism between environmental exposure (such as air pollution, noise) and depression [30, 31], and provided epidemiological evidence [32, 33], but the biological mechanism of extreme precipitation increasing the risk of depression outpatients is still unclear. Our findings suggested that extreme precipitation was associated with increased risk of the outpatient visits for depression. The possible explanation is as follows. Firstly, previous studies had found that environment stresses, such as cold or heat stress, which were caused by extreme weather events, were considered as risk factors for mental illness [34–36]. Secondly, extreme precipitation is accompanied by changes in sunlight and temperature. These changes may cause some mental diseases, which may be associated with the fluctuation of the serotonergic function [37]. A study has shown that the serotonergic varied inversely with daily temperature [38]. Furthermore, living in a warmer and sunnier climate may encourage people to go out and exercise more, which had been shown to be a protective factor for depression [39]. Last but not least, studies have reported the relationship between heat waves and mental disorders (such as depression and post-traumatic stress disorder), and indicated that it may increase the incidence of depression [26, 40, 41]. It is well known that heat wave is one of the reasons for precipitation, which explains our conclusion to some extent. Although some studies have shown the impact of extreme weather events on mental diseases [10, 42], further studies are needed to reveal the mechanism of extreme precipitation on depression.

In this subgroup analysis, we observed that females were more susceptible to the effect of extreme precipitation on depression than males, with higher rate of AF and AN. The possible reason was that, different from males, females, who possibly have strong stress responsiveness, often bear multiple care responsibilities for their children, spouses and parents, and have experienced more stressful life events [43]. These stressors may become risk factors for depression and eventually lead to depression [44]. And females who experienced pregnancy, breast-feeding, menstrual cycle and the menopausal transition were more likely to be affected by the outside environment and had relatively higher rates of depression [45]. Besides, people with multiple outpatient visits were more vulnerable to extreme precipitation than those with first outpatient visits. Some scholars had given the possible reason that most patients with depression have seasonal characteristics, and weather factors are one of the predisposing factors of recurrent depression [46]. Moreover, it indicated that extreme precipitation may have an increased effect for depression in the patients aged ≥19 years. Among them, people over 65 years were more vulnerable to extreme precipitation. This may be because with the increase of age, the physiological and psychological functions of the elderly are gradually getting weaken, especially the sensory organs and nervous systems involved in psychological activities may undergo degenerative changes. The physical resistance of the elderly is low, which is accompanied by a decrease in their ability to adapt to changes in the external environment [20]. Moreover, the elderly suffer from more chronic diseases, and the distress of these diseases may cause depression in the elderly. Therefore, the elderly were more sensitive to rainfall, which was consistent with previous research [47].

This study has several advantages. Firstly, to our knowledge, this maybe the first study to explore the association between extreme precipitation and depression by using a time series design. We comprehensively and deeply analyzed the RR, AF and AN with patients of depression caused by extreme precipitation, which provided important reference value for the prevention of depression in the region. Secondly, previous studies had reported that air pollutants may increase the risk of depression outpatient visits. Therefore, we added air pollutants into the model for sensitivity analysis to verify the robustness of the model, and finally showed that our results were robust. Thirdly, we used the DLNM to analyze the lag effect of extreme precipitation on depression, and through subgroup analysis, we found that the sensitive population of depression, suggesting that we should attach importance to susceptible population and protect them.

Nevertheless, some limitations should be taken into consideration in our study. Firstly, since we only selected a single city as the study area, this study may not be extended to other areas, especially those with different climates. Secondly, our meteorological data are derived from meteorological stations and therefore cannot accurately estimate the actual exposure of individuals. Thirdly, in this study, we only examined the short-term effects of extreme precipitation on depression, and future studies should explore this relationship in a longer time scale. Finally, in view of the differences in spatial distribution and obvious seasonality, the distribution of rainfall is abnormal and uneven. Therefore, the cut-off value of rainfall is not easy to obtain. In the future, more accurate and complex models may need to be established to simulate the exposure-response relationship.

## Conclusions

Our study found the adverse effect of extreme precipitation on depression outpatient visits from 2017 to 2019 in Suzhou, Anhui Province, China. Female, people aged ≥65 years and multiple outpatient visits for depression are more vulnerable to extreme precipitation. This study may enlighten relevant government departments to strengthen public health policy formulation and rationally allocate health resources. At the same time, more studies are needed to further confirm our results in other regions with the same climate type.

## Supplementary Information

**Additional file 1: Fig. S1.** Attributable fraction (AF) and numbers (AN) of extreme precipitation on outpatient visits for depression. **Fig. S2.** Sensitivity analysis when altering the degrees of freedom (*df* = 5–8) for controlling for the long-term trend and seasonality. **Fig. S3.** Sensitivity analysis when altering the degrees of freedom (*df* = 3–6) for controlling for mean temperature. **Fig. S4.** Sensitivity analysis when altering the degrees of freedom (*df* = 3–6) for controlling for relative humidity. **Fig. S5.** Sensitivity analysis when altering the degrees of freedom (*df* = 3–6) for controlling for the sunshine duration. **Fig. S6.** Relative risk and 95%CI of extreme precipitation on depression outpatient visits by adding other air pollutants in the sensitivity analysis. **Fig. S7.** Sensitivity analysis by changing the cut-off value of extreme precipitation in the model. **Table S1.** The AIC values of models for various lag period from lag1 to lag20. **Table S2.** The single-day effects of extreme precipitation on depression outpatient visits in different subgroups in Suzhou, China, with 95th percentile (13.13 mm) of precipitation relative to no precipitation. **Table S3.** Single-day and cumulative lag effects of extreme precipitation on depression outpatient visits at various lag days in Suzhou, China, with 95th percentile (13.13 mm) of precipitation relative to no precipitation.

## Data Availability

The datasets used and/or analysed during the current study are available from the corresponding author on reasonable request.

## Abbreviations

PM_2.5_: Particulate matter with aerodynamic diameter less than 2.5 μm
NO_2_: Nitrogen dioxide
SO_2_: Sulfur dioxide
AF: Attributable fraction
AN: Attributable number
ICD-10: International Classification of Diseases, the 10th version
DLNM: distributed lag non-linear model
SD: Standard deviation
RR: Relative risk
CI: Confidence interval
AIC: Akaike Information Criterion
