# Supplementary material for: Effects of extreme precipitation on hospital visit risk and disease burden of depression in Suzhou, China
Source: BMC Public Health. 2022 Sep 9;22:1710. doi: 10.1186/s12889-022-14085-w (PMC9463798; doi:10.1186/s12889-022-14085-w)
Supplement: Supplementary file 1 — Additional file 1: Fig. S1. Attributable fraction (AF) and numbers (AN) of extreme precipitation on outpatient visits for depression. Fig. S2. Sensitivity analysis when altering the degrees of freedom (df = 5–8) for controlling for the long-term trend and seasonality. Fig. S3. Sensitivity analysis when altering the degrees of freedom (df = 3–6) for controlling for mean temperature. Fig. S4. Sensitivity analysis when altering the degrees of freedom (df = 3–6) for controlling for relative humidity. Fig. S5. Sensitivity analysis when altering the degrees of freedom (df = 3–6) for controlling for the sunshine duration. Fig. S6. Relative risk and 95%CI of extreme precipitation on depression outpatient visits by adding other air pollutants in the sensitivity analysis. Fig. S7. Sensitivity analysis by changing the cut-off value of extreme precipitation in the model. Table S1. The AIC values of models for various lag period from lag1 to lag20. Table S2. The single-day effects of extreme precipitation on depression outpatient visits in different subgroups in Suzhou, China, with 95th percentile (13.13 mm) of precipitation relative to no precipitation. Table S3. Single-day and cumulative lag effects of extreme precipitation on depression outpatient visits at various lag days in Suzhou, China, with 95th percentile (13.13 mm) of precipitation relative to no precipitation. [file 12889_2022_14085_MOESM1_ESM.doc]

# Effects of extreme precipitation on hospital visit risk and disease burden of depression in Suzhou, China

Gang Jiang**^1†^**, Yanhu Ji**^2†^**, Changhao Chen^3^, Xiaosong Wang**^4^**, Tiantian Ye**^1^**, Yuhuan Ling**^1^**, Heng Wang**^1,4^,**^*^

**^1^**Department of Social Medicine and Health Management, School of Health Management, Anhui Medical University, Hefei, China

**^2^** Department of Epidemiology and Health Statistics, School of Public Health, Anhui Medical University, Hefei, China

**^3^** Department of Psychiatry, Suzhou Second People's Hospital, Suzhou, China

**^4^** The First Affiliated Hospital of Anhui Medical University, Hefei, China

* Correspondence: [wangheng1969@163.com](mailto:wangheng1969@163.com)

**^†^** Gang Jiang and Yanhu Ji contributed equally to this work.

**Supplementary materialFig S1** Attributable fraction (AF) and numbers (AN) of extreme precipitation on outpatient visits for depression

**Figure S2** Sensitivity analysis when altering the degrees of freedom (*df* = 5-8) for controlling for the long-term trend and seasonality

**Figure S3** Sensitivity analysis when altering the degrees of freedom (*df* = 3-6) for controlling for mean temperature

**Figure S4** Sensitivity analysis when altering the degrees of freedom (*df* = 3-6) for controlling for relative humidity

**Figure S5** Sensitivity analysis when altering the degrees of freedom (*df* = 3-6) for controlling for the sunshine duration

**Figure S6** Relative risk and 95%CI of extreme precipitation on depression outpatient visits by adding other air pollutants in the sensitivity analysis

**Figure S7** Sensitivity analysis by changing the cut-off value of extreme precipitation in the model

**Table S1** The AIC values of models for various lag period from lag1 to lag20.

**Table S2** The single-day effects of extreme precipitation on depression outpatient visits in different subgroups in Suzhou, China, with 95th percentile (13.13 mm) of precipitation relative to no precipitation

**Table S3** Single-day and cumulative lag effects of extreme precipitation on depression outpatient visits at various lag days in Suzhou, China, with 95th percentile (13.13 mm) of precipitation relative to no precipitation


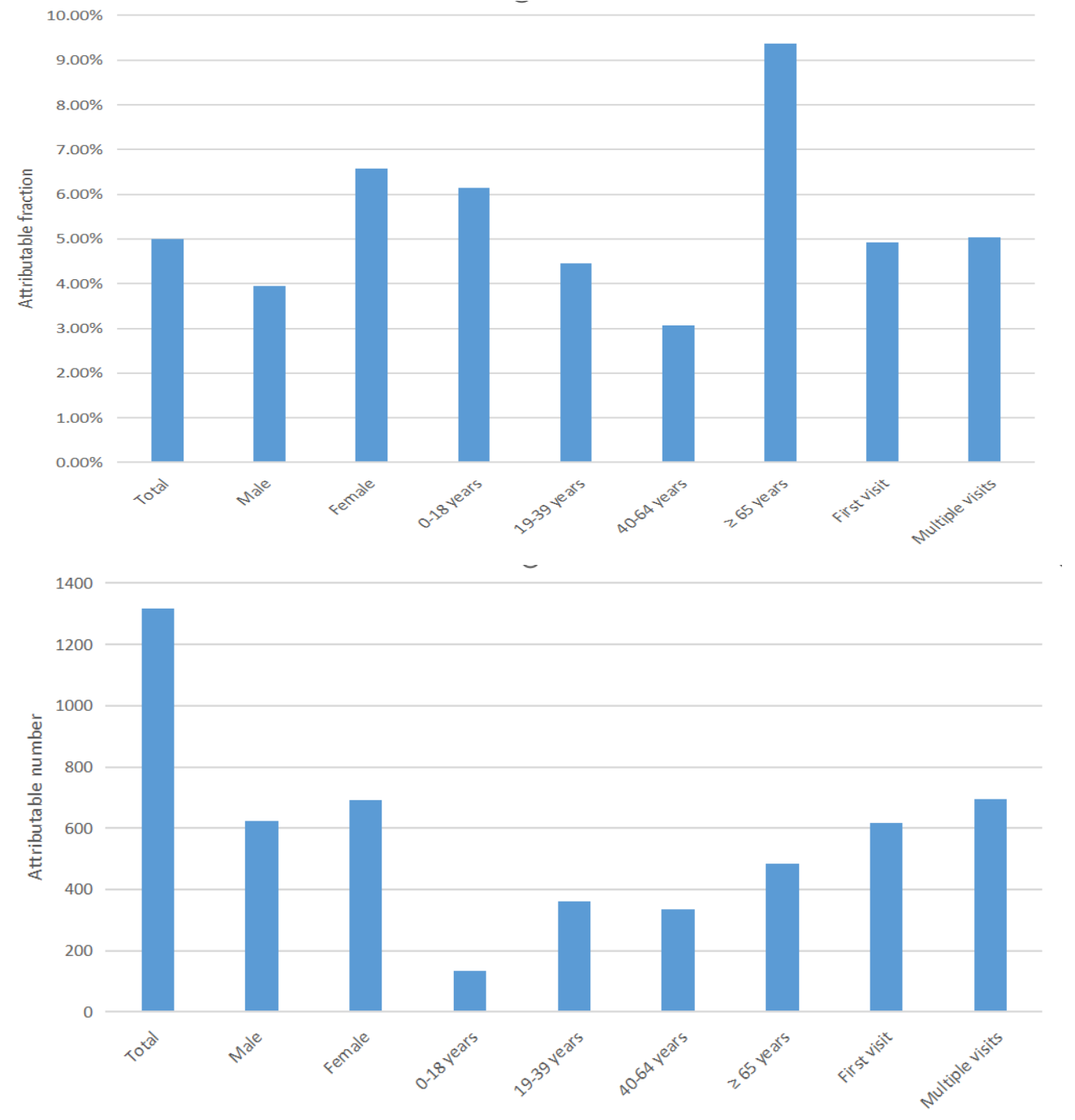


**Fig S1** Attributable fraction (AF) and numbers (AN) of extreme precipitation on outpatient visits for depression


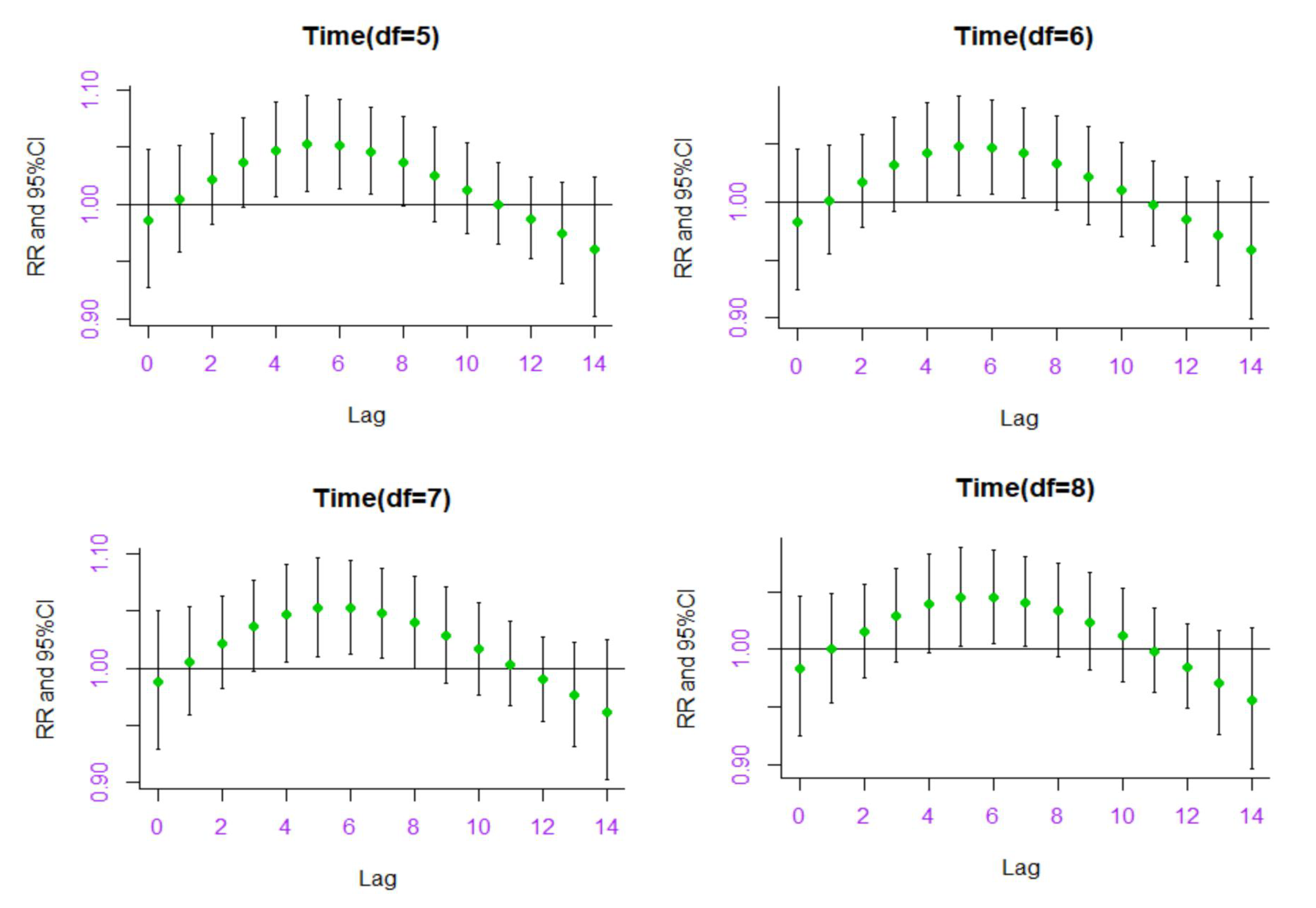


**Figure S2** Sensitivity analysis when altering the degrees of freedom (*df* = 5-8) for controlling for the long-term trend and seasonality


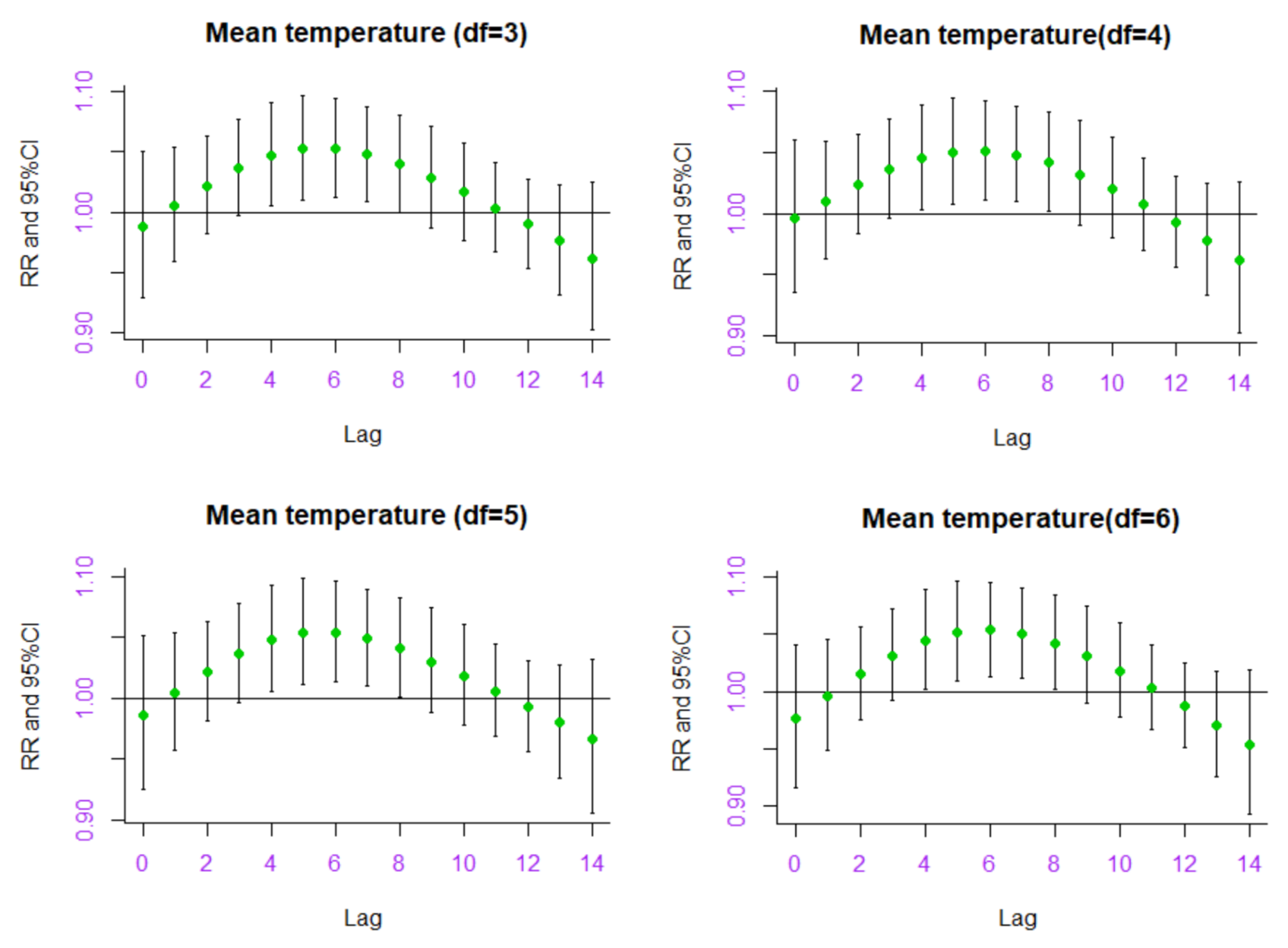


**Figure S3** Sensitivity analysis when altering the degrees of freedom (*df* = 3-6) for controlling for mean temperature


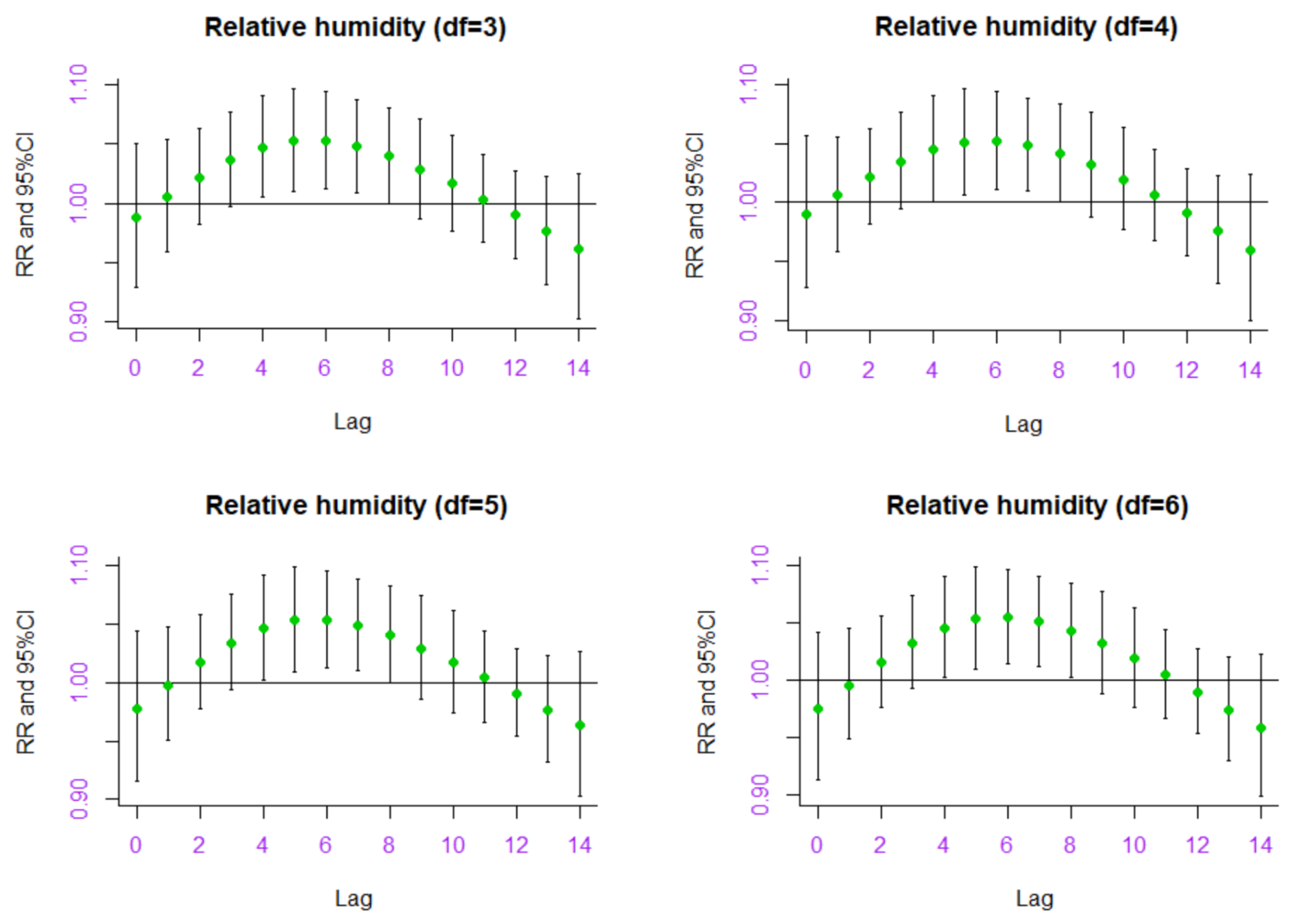


**Figure S4** Sensitivity analysis when altering the degrees of freedom (*df* = 3-6) for controlling for relative humidity


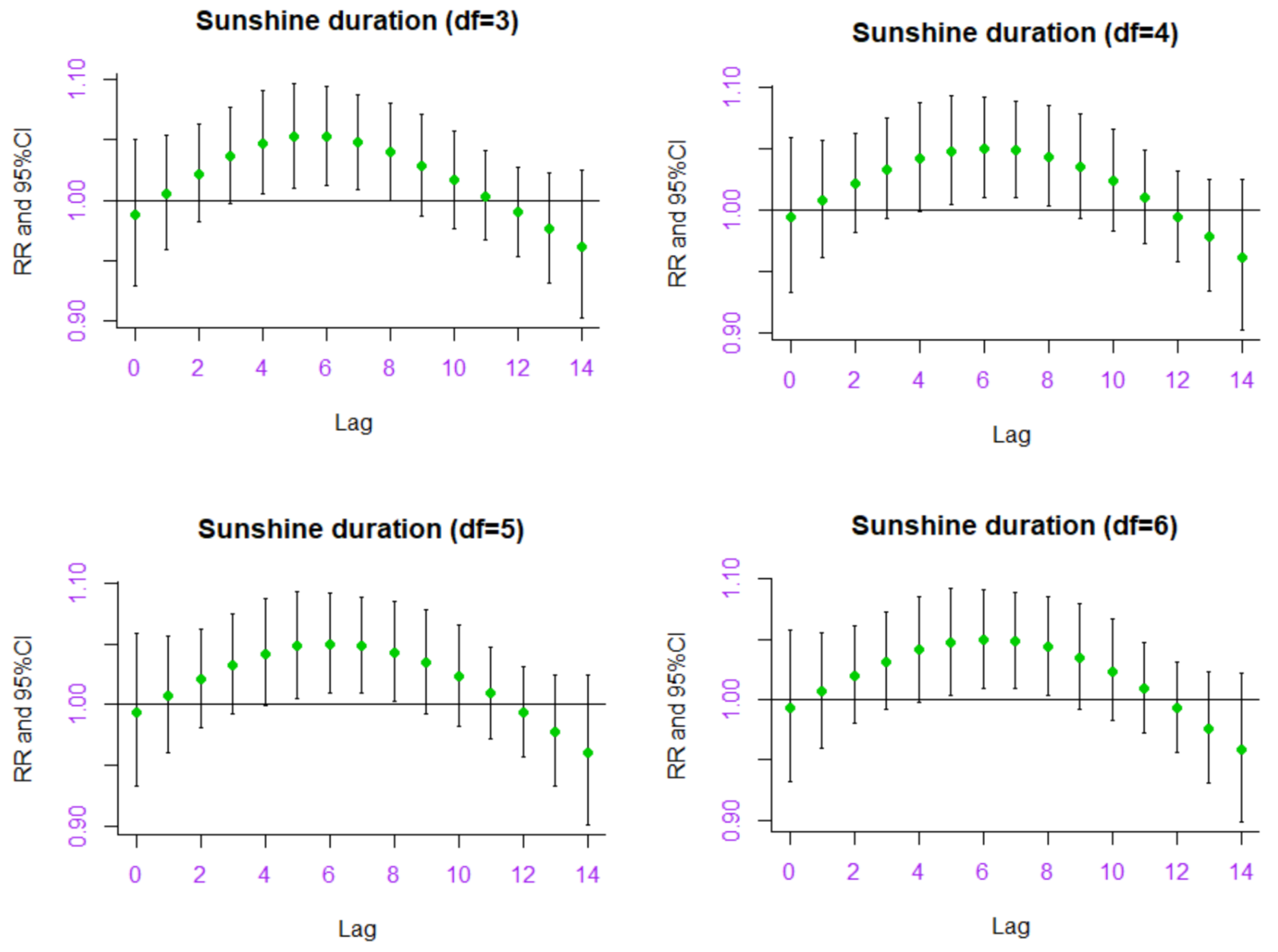


**Figure S5** Sensitivity analysis when altering the degrees of freedom (*df* = 3-6) for controlling for the sunshine duration


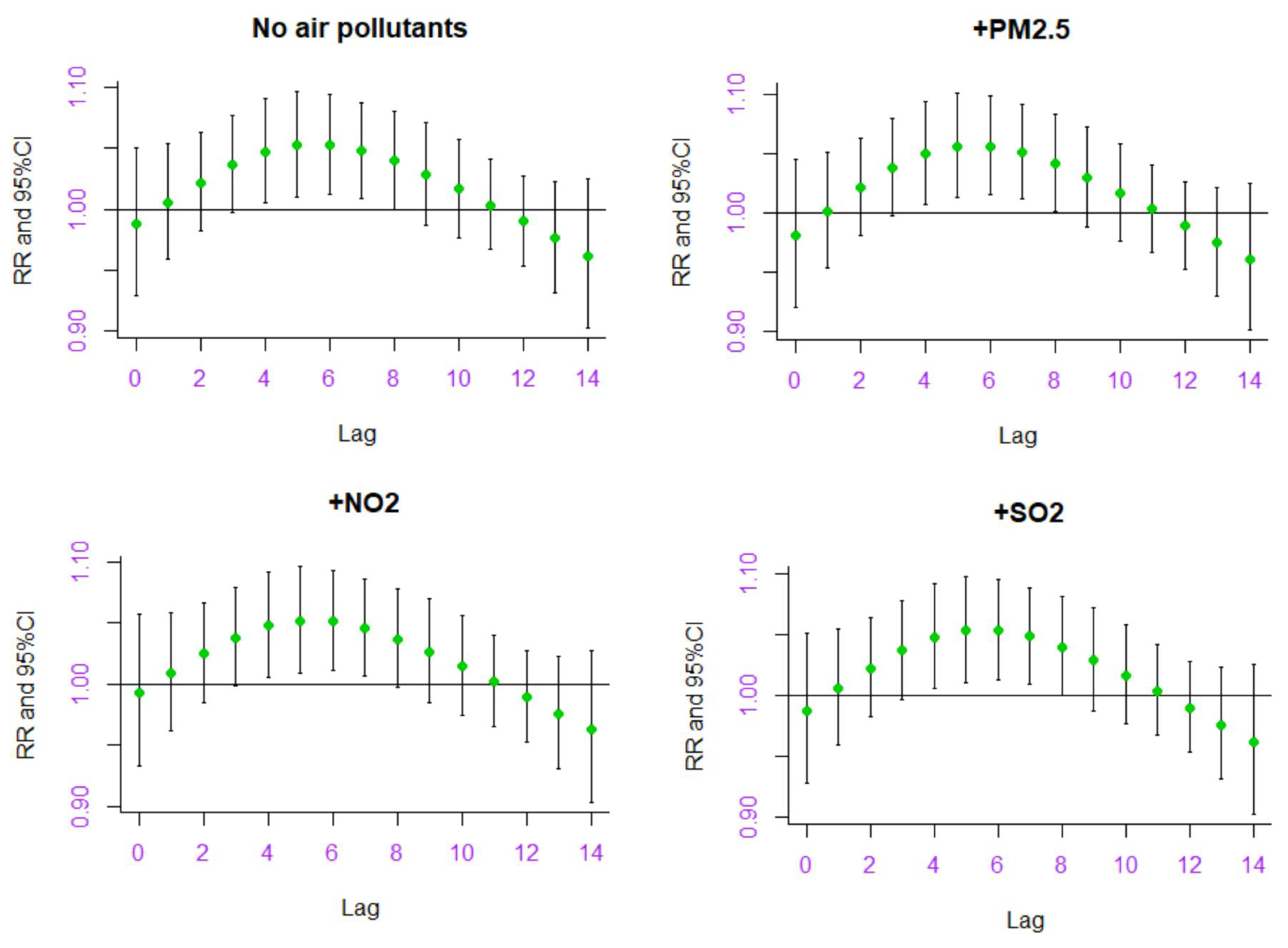


**Figure S6** Relative risk and 95%CI of extreme precipitation on depression outpatient visits by adding other air pollutants in the sensitivity analysis


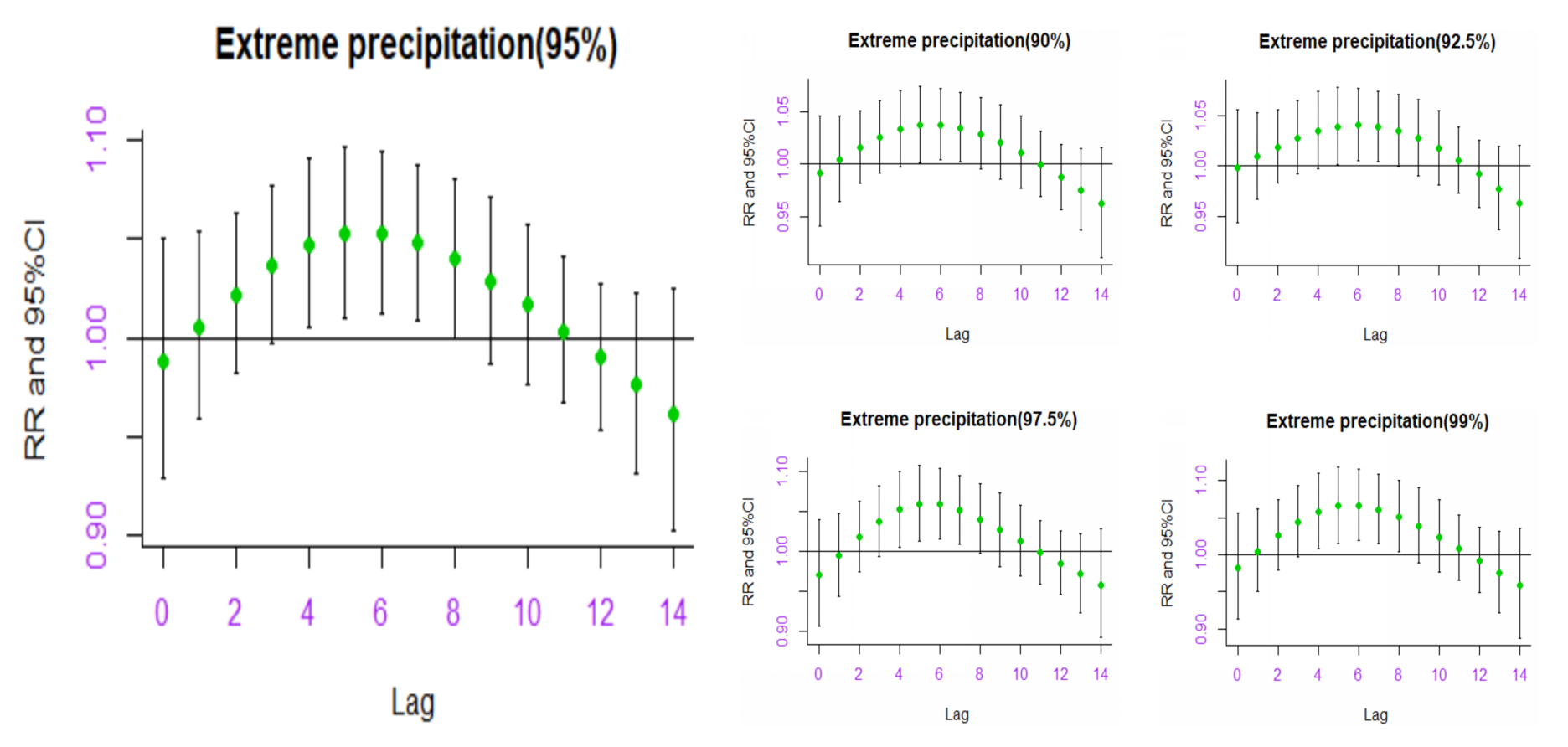


**Figure S7** Sensitivity analysis by changing the cut-off value of extreme precipitation in the model

**Table S1** The AIC values of models for various lag period from lag1 to lag20.

| Lag | Q-AIC | Lag | Q-AIC |
| --- | --- | --- | --- |
| 1 | 8450.962 | 11 | 8426.889 |
| 2 | 8448.664 | 12 | 8417.498 |
| 3 | 8448.051 | 13 | 8384.941 |
| 4 | 8445.380 | 14 | 8382.864 |
| 5 | 8440.097 | 15 | 8388.553 |
| 6 | 8439.079 | 16 | 8396.720 |
| 7 | 8438.454 | 17 | 8425.749 |
| 8 | 8436.486 | 18 | 8427.326 |
| 9 | 8430.894 | 19 | 8429.442 |
| 10 | 8428.507 | 20 | 8432.810 |

Table S2 The single-day effects of extreme precipitation on depression outpatient visits in different subgroups in Suzhou, China, with 95th percentile (13.13 mm) of precipitation relative to no precipitation

| Lag | Gender | | Age | | | | Visit type | |
| --- | --- | --- | --- | --- | --- | --- | --- | --- |
|  | Male | Female | 0-18 | 19-39 | 40-64 | ≥65 | First visit | Repeat visit |
| 0 | 0.994  (0.930-1.063) | 0.977  (0.900-1.061) | 0.984  (0.783-1.237) | 0.983  (0.920-1.050) | 0.972  (0.926-1.020) | 1.027  (0.902-1.169) | 0.996  (0.915-1.085) | 0.980  (0.920-1.043) |
| 1 | 1.009  (0.958-1.061) | 1.001  (0.939-1.065) | 1.005  (0.842-1.198) | 0.999  (0.949-1.051) | 0.987  (0.951-1.024) | 1.053  (0.953-1.163) | 1.013  (0.949-1.082) | 0.998  (0.951-1.047) |
| 2 | 1.022  (0.979-1.066) | 1.022  (0.970-1.077) | 1.025  (0.883-1.189) | 1.014  (0.971-1.058) | 1.002  (0.971-1.033) | 1.076  (0.990-1.170) | 1.029  (0.974-1.087) | 1.015  (0.975-1.057) |
| 3 | 1.032  (0.990-1.077) | 1.042  (0.989-1.098) | 1.042  (0.899-1.208) | 1.027  (0.985-1.071) | 1.014  (0.984-1.046) | 1.095  (1.008-1.189)* | 1.042  (0.987-1.100) | 1.031  (0.991-1.072) |
| 4 | 1.039  (0.994-1.086) | 1.059  (1.002-1.118)* | 1.056  (0.904-1.234) | 1.038  (0.993-1.085) | 1.025  (0.992-1.058) | 1.105  (1.012-1.205)* | 1.050  (0.992-1.111) | 1.044  (1.001-1.088)* |
| 5 | 1.041  (0.995-1.088) | 1.070  (1.013-1.130)* | 1.065  (0.912-1.244) | 1.046  (1.001-1.094)* | 1.031  (0.999-1.065) | 1.103  (1.011-1.204)* | 1.051  (0.993-1.113) | 1.053  (1.010-1.097)* |
| 6 | 1.037  (0.994-1.081) | 1.076  (1.022-1.133)* | 1.068  (0.922-1.236) | 1.050  (1.007-1.095)* | 1.034  (1.003-1.065)* | 1.089  (1.003-1.183)* | 1.046  (0.991-1.104) | 1.057  (1.016-1.099)* |
| 7 | 1.029  (0.988-1.071) | 1.076  (1.023-1.131)* | 1.064  (0.923-1.226) | 1.050  (1.008-1.093)* | 1.032  (1.003-1.063)* | 1.068  (0.986-1.157) | 1.036  (0.983-1.091) | 1.057  (1.018-1.098)* |
| 8 | 1.018  (0.976-1.063) | 1.070  (1.016-1.127)* | 1.053  (0.908-1.222) | 1.047  (1.004-1.092)* | 1.028  (0.998-1.060) | 1.042  (0.959-1.133) | 1.023  (0.969-1.080) | 1.053  (1.012-1.095)* |
| 9 | 1.007  (0.963-1.053) | 1.060  (1.004-1.120)* | 1.036  (0.886-1.212) | 1.041  (0.996-1.088) | 1.022  (0.990-1.055) | 1.017  (0.931-1.110) | 1.009  (0.953-1.068) | 1.045  (1.002-1.089)* |
| 10 | 0.997  (0.954-1.041) | 1.045  (0.992-1.103) | 1.012  (0.868-1.179) | 1.033  (0.989-1.078) | 1.014  (0.983-1.046) | 0.994  (0.912-1.082) | 0.995  (0.941-1.052) | 1.034  (0.993-1.077) |
| 11 | 0.987  (0.948-1.027) | 1.027  (0.978-1.079) | 0.983  (0.854-1.131) | 1.022  (0.983-1.063) | 1.005  (0.977-1.034) | 0.974  (0.901-1.054) | 0.983  (0.934-1.034) | 1.020  (0.983-1.059) |
| 12 | 0.978  (0.939-1.018) | 1.007  (0.958-1.058) | 0.950  (0.824-1.096) | 1.011  (0.971-1.052) | 0.995  (0.967-1.024) | 0.957  (0.885-1.036) | 0.971  (0.922-1.022) | 1.005  (0.967-1.043) |
| 13 | 0.969  (0.921-1.019) | 0.985  (0.925-1.048) | 0.916  (0.767-1.095) | 0.998  (0.949-1.050) | 0.985  (0.950-1.021) | 0.942  (0.854-1.040) | 0.960  (0.900-1.024) | 0.988  (0.942-1.036) |
| 14 | 0.961  (0.897-1.030) | 0.962  (0.884-1.048) | 0.882  (0.692-1.123) | 0.985  (0.920-1.055) | 0.974  (0.927-1.024) | 0.928  (0.811-1.063) | 0.949  (0.869-1.037) | 0.971  (0.910-1.036) |

* *P*<0.05

**Table S3** Single-day and cumulative lag effects of extreme precipitation on depression outpatient visits at various lag days in Suzhou, China, with 95th percentile (13.13 mm) of precipitation relative to no precipitation

| Single-day | Single effects RR (95% CI) | Multi-day | Cumulative effects RR (95% CI) |
| --- | --- | --- | --- |
| 0 | 0.987(0.928-1.050) | 0-0 | 0.987(0.928-1.050) |
| 1 | 1.005(0.959-1.053) | 0-1 | 1.021(0.917-1.137) |
| 2 | 1.022(0.982-1.063) | 0-2 | 1.053(0.914-1.214) |
| 3 | 1.036(0.996-1.077) | 0-3 | 1.099(0.927-1.304) |
| 4 | 1.047(1.005-1.091)* | 0-4 | 1.156(0.949-1.407) |
| 5 | 1.052(1.010-1.096)* | 0-5 | 1.219(0.975-1.522) |
| 6 | 1.052(1.012-1.094)* | 0-6 | 1.282(1.002-1.641)* |
| 7 | 1.047(1.009-1.087)* | 0-7 | 1.341(1.025-1.754)* |
| 8 | 1.039(0.999-1.080) | 0-8 | 1.391(1.042-1.855)* |
| 9 | 1.028(0.987-1.071) | 0-9 | 1.427(1.049-1.941)* |
| 10 | 1.016(0.976-1.058) | 0-10 | 1.448(1.044-2.009)* |
| 11 | 1.003(0.967-1.040) | 0-11 | 1.455(1.029-2.055)* |
| 12 | 0.989(0.953-1.027) | 0-12 | 1.445(1.006-2.076)* |
| 13 | 0.975(0.931-1.022) | 0-13 | 1.420(0.973-2.074) |
| 14 | 0.962(0.902-1.025) | 0-14 | 1.381(0.927-2.058) |

* *P*<0.05
